# Plastics as a Bar to Bacteria

**Published:** 1895-06

**Authors:** Joseph Head

**Affiliations:** Philadelphia


### PLASTICS AS A BAR TO BACTERIA.[1]

BY JOSEPH HEAD, M.D., D.D.S., PHILADELPHIA.

"THE perfect filling must be water-tight," so says the time-honored axiom.

But a tooth is everywhere permeable by moisture, as can readily be demonstrated by dropping it, when dry, into aniline ink. It will be stained through and through.

One can hope to put a water-tight plug into a wet sponge quite as readily as into tooth-structure that is moist throughout. Thus we find that neither the axiom nor the filling will hold water.

Should we not rather say that, though a filling leak, it may still be perfect if it exclude germs of decay?

---

[1] Read before the Odontological Society of Pennsylvania, January 12, 1895.

On superficial observation it might be thought that wherever moisture could penetrate so could bacteria; but this does not seem to be the case, as bacteria have not yet been found in normal dental tubules.

Before decay can take place some free acid must first decalcify the enamel and the orifice of a tubule. In this enlarged opening the bacteria may enter, when devouring the gelatin it excretes acid, which, dissolving more of the lime salts, allows the germ to penetrate farther in (Sudduth).

The ideal filling would be composed of a material that is water-proof, bacteria-proof, adherent, and a non-conductor of heat. And, moreover, a material that can readily be placed in the cavity without the slightest danger of marring the enamel edges.

With such a filling in position, the tooth would be as likely to decay as it was before decalcification occurred.

But we have not yet found the ideal filling-material.

Gold, while it can be made to exclude bacteria from its own substance and from entrance at the edges of the filling, is a good conductor of heat, and can only be sufficiently hammered to make a perfect seal in teeth of dense structure. There is no doubt that gold can be made water-proof, as the Bonwill mallet has demonstrated time and time again. Teeth have been partially contoured one day and completed the next, perfect welding having been accomplished after the thinnest film of the moistened gold has been removed. But soft-foil fillings that have such a record for preserving teeth have been put in under water with lasting results. Does this filling preserve the teeth by starving out the micro-organisms? This supposition can hardly hold, as soft-foil fillings have been removed from cavities in a punky, evil-smelling state by means of the explorer, and the dentine beneath appeared sound and dense.

Tin would seem pervious to moisture, as any old filling can readily be separated into portions that only partially adhere. It has not been proved, one way or the other, whether it admits bacteria; but at least it can be said to preserve tooth-structure in a manner very similar to soft foil.

Amalgam in itself is water-proof, but leaks at the cavity margins.

Each year we hear of some wonderful alloy that will not shrink from the walls; but, in my opinion, that amalgam has not yet been discovered.

Reducing the mercury to a minimum will do much, but it has been my experience that amalgam fillings of my own and those of my fellow-practitioners all show some slight shrinking from the enamel after the space of some five or six years. But even granting that all these fillings had been badly put in, we still find, when the bulging edges have been trimmed, that in spite of a palpable leak in many instances the cavity remained sound. It may be affirmed that metallic rust gets into the tubules and protects them. That is certainly some sort of an explanation, and yet it can hardly be accepted as conclusive.

Oxychloride of zinc and oxyphosphate of zinc, although adherent to the tooth walls, are readily penetrable by moisture and bacteria.

Gutta-percha, although practically if not absolutely impervious to moisture, invariably leaks at the cavity margins.

My experiments with gold, tin, and amalgam are not yet completed, and so it is possible only to deduce from facts that are clinically well known; but the assertions just made concerning the cements and gutta-percha seem to have been proved in the following manner.

Cones of oxychloride of zinc and oxyphosphate of zinc were made, having a hollow place within that was absolutely excluded from the outer air. Harvard and Peirce's oxyphosphate of zinc was used that became hard as ivory. Some of the oxychloride of zinc cones were made from calcined powder, some from the uncalcined. Those made from the calcined powder became extremely dense.

These cones were sterilized in a steam bath by the intermittent process in the following way: First, they were boiled in water two to three hours; then removed from the bath and placed in glass jars, the mouths of which were closed with absorbent cotton, and subjected to steam over the water-bath for one hour.

They were then allowed to cool for seven hours in an atmosphere of about 70° F.; again subjected to steam for one hour, and cooled over night. Next morning again heated for about an hour. Allowed to cool eight hours. Four hours steam heat, twenty-four hours to cool; one hour steam heat, allowed to cool, and then placed in a bath that after being first sterilized had been tainted with a decayed tooth. At the end of five days' immersion they were taken out and opened. The bouillon had filtered through the substance to the hollows inside. The bouillon found within was swarming with micro-organism.

The steam bath did not have the slightest effect on the oxychloride of zinc, but the oxyphosphate, from being very dense, seemed much softened. This might seem to depreciate from the value of that particular experiment; but it would still seem probable, if the micrococci could pass through strong oxychloride, that they could also pass through the oxyphosphate, that is so similar to it in substance.

On drying the opened oxyphosphate cones, small, shiny crystals lined the inside, which looked not unlike free phosphoric acid that had not become chemically united with the powder. And yet the cones before boiling were extremely dense.

The experiments with gutta-percha were as follows: Three canine teeth were taken and opened from end to end. The surfaces of the canals were thoroughly drilled away. One end of each was filled firmly with gutta-percha. A small pellet of cotton soaked with sterilized broth was then placed in the canal. The remaining openings were then dried and filled with gutta-percha. These were sterilized as follows: Two and a half hours in steam bath, four hours to cool, one hour in boiling water, two hours in steam bath, seven hours to cool, two hours in bath, two hours to cool, and placed in tainted broth for five days.

At the end of that time they were taken out, dried, and passed rapidly once or twice through a Bunsen flame. The gutta-percha was then removed with a heated instrument, the cotton was taken out by tweezers previously sterilized in the flame, placed on a clean glass slide, and wet with two drops of distilled water. This water was then found to contain large numbers of bacteria.

If these facts are so, and the evidence would seem to indicate that they are so, what becomes of the fundamental principle involved in the statement that the edges of a filling must be bacteria-proof.

We know that cohesive gold fillings are almost if not quite certain to admit decay if the edges are not thoroughly tight. Then necessity for bacteria exclusion would seem not to entirely hold in the case of soft foil.

It may or may not be necessary with tin. It certainly is not absolutely imperative in the case of amalgam. With cement and gutta-percha it also does not seem to be an essential to tooth preservation.

And yet all of these filling-materials save teeth, and save them well. Especially is it the case with cement and gutta-percha, that stop decay when nothing else will. These last preserve the cavity walls, and yet allow the bacteria to enter. This seems a paradox, and is difficult to explain. One might say that bacteria need air and food; that cement and gutta-percha shut the germs off from these necessities, and thus render them dormant. But this does not to my mind reveal why some soft, spongy, malodorous soft-foil fillings have preserved the dentine beneath from further decay.

The experiments just reported may seem to be a means of deducing new and startling facts, but in reality this is not so. Soft foil and amalgam foreshadowed these conclusions many years ago.

I should like to add one word for those who shall ever wish to sterilize teeth for bacteriological research. Do not do it in a vulcanizer under steam pressure. I placed fifteen teeth carefully prepared in a vulcanizer, and kept them there for one hour at a pressure of thirty pounds, temperature 260° F.

When they were removed almost all the albuminous material had been extracted. They would have served as excellent specimens wherein college students might easily and readily examine the position and size of the canals, but were hardly suitable for bacteriological experiments.

In closing I would express my thanks to Miss Byrnes, the Fellow of Biology at Bryn Mawr, who not only placed the requisite apparatus at my disposal, but also gave valuable advice and assistance.

